# Evaluation of Computational Docking to Identify Pregnane X Receptor Agonists in the ToxCast Database

**DOI:** 10.1289/ehp.1001930

**Published:** 2010-06-17

**Authors:** Sandhya Kortagere, Matthew D. Krasowski, Erica J. Reschly, Madhukumar Venkatesh, Sridhar Mani, Sean Ekins

**Affiliations:** 1 Department of Microbiology and Immunology, Drexel University College of Medicine, Philadelphia, Pennsylvania, USA; 2 Department of Pathology, University of Pittsburgh, Pittsburgh, Pennsylvania, USA; 3 Albert Einstein Cancer Center, Albert Einstein College of Medicine, Bronx, New York, USA; 4 Collaborations in Chemistry, Jenkintown, Pennsylvania, USA; 5 Department of Pharmaceutical Sciences, University of Maryland, Baltimore, Maryland, USA; 6 Department of Pharmacology, Robert Wood Johnson Medical School, University of Medicine and Dentistry of New Jersey, Piscataway, New Jersey, USA

**Keywords:** Bayesian model, docking, GoldScore, hybrid scoring, PXR, ToxCast

## Abstract

**Background:**

The pregnane X receptor (PXR) is a key transcriptional regulator of many genes [e.g., cytochrome P450s (*CYP2C9, CYP3A4, CYP2B6*), *MDR1*] involved in xenobiotic metabolism and excretion.

**Objectives:**

As part of an evaluation of different approaches to predict compound affinity for nuclear hormone receptors, we used the molecular docking program GOLD and a hybrid scoring scheme based on similarity weighted GoldScores to predict potential PXR agonists in the ToxCast database of pesticides and other industrial chemicals. We present some of the limitations of different *in vitro* systems, as well as docking and ligand-based computational models.

**Methods:**

Each ToxCast compound was docked into the five published crystallographic structures of human PXR (hPXR), and 15 compounds were selected based on their consensus docking scores for testing. In addition, we used a Bayesian model to classify the ToxCast compounds into PXR agonists and nonagonists. hPXR activation was determined by luciferase-based reporter assays in the HepG2 and DPX-2 human liver cell lines.

**Results:**

We tested 11 compounds, of which 6 were strong agonists and 2 had weak agonist activity. Docking results of additional compounds were compared with data reported in the literature. The prediction sensitivity of PXR agonists in our sample ToxCast data set (*n =* 28) using docking and the GoldScore was higher than with the hybrid score at 66.7%. The prediction sensitivity for PXR agonists using GoldScore for the entire ToxCast data set (*n* = 308) compared with data from the NIH (National Institutes of Health) Chemical Genomics Center data was 73.8%.

**Conclusions:**

Docking and the GoldScore may be useful for prioritizing large data sets prior to *in vitro* testing with good sensitivity across the sample and entire ToxCast data set for hPXR agonists.

The pregnane X receptor (PXR, also known as NR1I2, SXR, or PAR) is a nuclear hormone receptor (NHR) that regulates the transcription of genes involved in xenobiotic metabolism and excretion. PXR agonists include a wide range of structurally diverse endogenous and exogenous compounds such as bile acids, steroid hormones, dietary fat-soluble vitamins, prescription medications, and herbal drugs, as well as environmental chemicals such as pesticides, estrogens, and antiestrogens (Mnif et al. 2007). PXR agonists can mediate clinically significant drug–drug interactions (Ekins et al. 2007, 2008a; Mani et al. 2009). Furthermore, PXR action in various pathophysiological states indicates that PXR agonists could variably affect human and animal health. For example PXR agonists can impact cholesterol metabolism and the endocrine system (Wada et al. 2009; Zhai et al. 2007) as well as potentiate the toxicity of other environmental contaminants, as reviewed recently (Biswas et al. 2009). Animal models may not reliably predict human PXR (hPXR)-related problems because of the diversity of PXRs across species (Ekins et al. 2008b; Moore et al. 2002), resulting in differences in ligand selectivity (Tirona et al. 2004). Therefore, the identification and characterization of hPXR agonists is important to human pharmacokinetics and toxicology of environmental chemicals.

Five hPXR crystal structures (1M13, 1NRL, 1SKX, 2O9I, and 2QNV) are available in the Protein Data Bank (PDB) (Chrencik et al. 2005; Teotico et al. 2008; Watkins et al. 2003a, 2003b; Xue et al. 2007a), with another structure to be deposited (Xue et al. 2007b). These structures have enabled characterization of the ligand-binding domain (LBD) and PXR–ligand interactions. The cocrystallized ligands tend to be hydrophobic, with a wide range in shape, size, and chemical composition that can be accommodated in the LBD (Ekins et al. 2009). The hPXR ligand-binding pocket (LBP) is lined with 28 amino acid residues: 20 hydrophobic, 4 polar, and 4 charged. Because of the large size and flexibility of the LBP, molecules can bind in multiple locations. This creates a challenge for *in silico* methods for predicting hPXR agonists, but many approaches have been evaluated nonetheless (Ekins et al. 2009). An added complexity to predicting whether a compound binds to PXR is the recent discovery that some molecules can also bind outside the LBD on the PXR surface and act as allosteric antagonists (Ekins et al. 2007, 2008a). It is also difficult to computationally identify antagonists that would compete with agonist binding in the LBD (Xue et al. 2007a).

Several previous studies have constructed ligand-based computational models for hPXR agonists employing pharmacophores (Bachmann et al. 2004; Ekins and Erickson 2002; Ekins et al. 2007; Schuster and Langer 2005), quantitative structure–activity relationships (QSARs), and machine learning methods (Ekins et al. 2006, 2009; Jacobs 2004; Ung et al. 2007; Yasuda et al. 2008). hPXR agonist pharmacophore models have been shown to possess hydrophobic, hydrogen bond acceptor, and hydrogen bond donor features, a finding consistent with the crystallographic structures of hPXR ligand-receptor complexes (Bachmann et al. 2004; Ekins et al. 2007; Ekins and Erickson 2002; Schuster and Langer 2005). These pharmacophore features may also relate closely to a recent analysis in which Ngan et al. (2009) used docking of small probe molecules into the LBD to identify five hot spots. As part of an ongoing analysis of NHRs (Ekins et al. 2008b; Reschly and Krasowski 2006; Reschly et al. 2007, 2008a, 2008b), we recently generated a large volume of experimental data for classes of steroidal compounds (Ekins et al. 2008b) and used it to evaluate various modeling approaches such as Bayesian classification modeling with 2-dimensional (2D) fingerprints, various QSAR approaches, and molecular docking into the available hPXR crystal structures (Ekins et al. 2009). Docking coupled with hybrid scoring 5D-QSAR methods performed significantly better than other QSAR methods in identifying agonists among these steroidal ligands (Ekins et al. 2009). With a promiscuous protein such as PXR, it is probably important to have global models or methods that can make predictions for a structurally diverse array of molecules rather than for a narrow structural series.

In previous studies we used structure-based docking, employing FlexX (BioSolveIT GmbH, Sankt Augustin, Germany) combined with logistic regression (Khandelwal et al. 2008), and GoldScore (Cambridge Crystallographic Data Centre, Cambridge, UK) combined with other descriptors as a weighting factor (Kortagere et al. 2009). Both FlexX and GOLD had mixed success in predicting a large set of structurally diverse hPXR agonists (Khandelwal et al. 2008; Kortagere et al. 2009), possibly because of the size and flexibility of the LBP, as described above (Ekins et al. 2009).

ToxCast represents a major U.S. Environmental Protection Agency (U.S. EPA) initiative for prioritizing the timely toxicity testing of large numbers of pesticides and other industrial chemicals (Dix et al. 2007; Houck et al. 2009; Judson et al. 2009, 2010; Knight et al. 2009) that may indicate various toxicity end points. In this study we initially used docking and scoring approaches to classify the hPXR agonist activity of these ToxCast compounds and prioritized them for *in vitro* screening prior to release of U.S. EPA experimental data. Our aim was to select a small subset of the compounds for testing to show that we could readily identify PXR agonists and PXR nonagonists without the need for screening all the compounds *in vitro*. We have also used the ToxCast data set to further evaluate whether docking coupled with a hybrid scoring scheme was useful as a predictive method for PXR, especially when screening large data sets of molecules. Although most ToxCast compounds are pesticides or other industrial chemicals, in this case there may be some overlap with the chemical space of pharmaceuticals, making this data set of interest for general PXR-agonist prediction. In addition, for comparison and to illustrate the difficulties of using local ligand-based models, we used a recently generated Bayesian model with 115 steroidal PXR agonists and nonagonists (Ekins et al. 2009) to classify the ToxCast compounds. While the present study was in progress, the ToxCast initiative generated data on all the compounds from the NIH (National Institutes of Health) Chemical Genomics Center (NCGC) (Judson et al. 2010). Therefore, we evaluated all available ToxCast hPXR *in vitro* data for these compounds using these computational methods to make predictions.

## Materials and Methods

### Materials

We purchased the DPX-2 cell line and the corresponding dosing and culturing medium from Puracyp Inc. (Carlsbad, CA). The creation of a HepG2 (human liver) cell line stably expressing the human Na^+^-taurocholate cotransporter (NTCP) has been previously reported and described in detail (Krasowski et al. 2005b). For tissue culture, we obtained BD Falcon Petri dishes from BD Biosciences (San Jose, CA); opaque treated sterile white 96-well assay plates with lids, and flat-bottom treated sterile (white with clear bottom) assay plates from Corning Inc. (Corning, NY). Disposable sterile pipette tips (low-adhesion tips) were purchased from BioTek (Winooski, VT), and the CellTiter-Fluor and Bright Glow assay system from Promega (Madison, WI). We used the Synergy Mx monochromator- based multimode microplate reader, EL406 Combination Washer Dispenser, and Precision XS microplate sample processor (BioTeK) for cell plating, drug dilution and plating, and reagent addition.

### Docking and scoring

We obtained chemical structures of the ToxCast molecules from the U.S. EPA Distributed Structure-Searchable Toxicity Database Network web site (U.S. EPA 2009); these structures were docked into the five crystal structures of hPXR (1M13, 1NRL, 1SKX, 2O9I, and 2QNV) using the docking program GOLD (version 4) as described by Jones et al. (1997). GOLD uses a genetic algorithm to explore the various conformations of ligands and flexible receptor side chains in the LBP. We performed 20 independent docking runs for each ligand, and the complexes were scored using GoldScore. In all cases before now, the crystal structure ligand was removed, and hydrogen atoms were added to the amino acids. All amino acids within 6 Å of the cocrystallized ligand were identified as the binding site.

From the entire set of ToxCast molecules that were docked to all five crystal structures, we chose 13 high-scoring and 2 low-scoring compounds to form the sample set. In addition, 13 compounds reported by Lemaire et al. (2006)—that docked to the five crystal structures and were scored using GoldScore—were added to our sample set. For all 28 compounds in the sample data set, we performed the GoldScore-based classification by choosing 80% of the GoldScore of the corresponding cocrystal ligand as a cutoff for whether a compound was an agonist (Table 1). The complexes were also scored using a hybrid scoring scheme, which was designed as GoldScore weighted with similarity scores (Kortagere et al. 2009). The similarity scores were based on 2D similarity (Sheridan et al. 2004; Willett 2003) encoded in MDL (Molecular Design Limited) fingerprint keys calculated using Discovery Studio 2.1 (Accelrys, San Diego, CA). The Tanimoto coefficient was used as the metric to compare the resulting molecular fingerprints. The coefficients varied between 0 (dissimilar) and 1 (similar) and were computed for all 28 compounds, with reference to the five cocrystal structure ligands (HYF, SRL, RFP, 444, and CDZ). Further, the weighted docking score of an active compound *i* with *j* conformations can be computed by


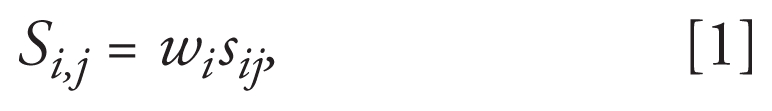


where *s_ij_* is the original GoldScore for the compound *i* in its *j*th conformation and *w_i_* is the similarity score for compound *i* with respect to the cocrystal structure ligand. For each of the 28 compounds, we calculated the average weighted score across all five crystal structures [see Supplemental Material, Table 1 (doi:10.1289/ehp.1001930)], and this score (average *S_ij_* ~ 15) was used as a cutoff for the classification. The GoldScores of the compounds and their similarity and weighted scores and classification are listed in Supplemental Material, Table 1. The consensus classification prediction was based on the majority vote across the five structures for each molecule. We classified complexes of all compounds from the ToxCast database with the five crystal structures using the GoldScore and hybrid score as described for the sample data set. We computed an average docking score and an average hybrid score for each crystal structure and used these as cutoff scores for the GoldScore- and hybrid score–based classifications, respectively (Table 1).

Predictions based on different docking methods were assessed using a standard set of statistical indicators to evaluate different ligand and docking classification approaches: sensitivity, specificity, overall prediction accuracy, and Matthews correlation coefficient (Kortagere et al. 2009). We generated 2D schematic representations of the ligands in the binding site using the LIGX option in MOE (Chemical Computing Group, Montreal, Quebec, Canada).

### Machine learning with 2D descriptors

We generated a Bayesian classification model using Discovery Studio 2.1 with the Laplacian-corrected Bayesian classifier and molecular descriptors, as previously described for 115 steroidal compounds (namely, androstanes, estratrienes, pregnanes, and bile salts), with hPXR activation determined by a luciferase-based reporter assay (Ekins et al. 2009). Compounds with Bayesian scores above −5.792 were classed as PXR agonists (Ekins et al. 2009).

### Reporter gene assay with HepG2 cells

The 13 top-scoring compounds and 2 low-scoring compounds predicted as nonagonists by GoldScore were selected for *in vitro* testing in the HepG2 human liver cell line. We determined hPXR activation in HepG2 cells using a luciferase-based reporter assay, as previously described (Ekins et al. 2008b; Krasowski et al. 2005a). Ligands that activated hPXR were classified as strong [median effective concentration (EC_50_) < 10 μM], medium (EC_50_ = 11–50 μM), or weak (EC_50_ > 50 μM, but still able to activate with at least 10% of the efficacy of 10 μM rifampicin) agonists (Ekins et al. 2008b).

#### Reporter gene assay with DPX-2 cells

The tissue culture protocols were performed in a sterile laminar flow hood, and all incubations were carried out at 37°C and 5% CO_2_. When DPX-2 cells were approximately 50–70% confluent, medium was aspirated and cells rinsed with 5 mL phosphate-buffered saline (PBS). The PBS was replaced with 2 mL trypsin/EDTA and incubated for 5 min. Two milliliters medium was added, and the entire mixture was transferred to a centrifuge tube. Cells were centrifuged at 900 rpm for 3 min and resuspended in 5 mL culturing medium; 100 μL cell suspension (corresponding to 25,000 cells) was added to each well of a 96-well plate, using an eight-channel Precision XS microplate sample processor, and incubated overnight. Drug stock solutions (50 mM for each compound) were prepared in DMSO and diluted to 11-point 1:3 compound titrations (50–0 μM) using the Precision XS to serially transfer 75 μL of diluted compounds into 150 μL Puracyp dosing media. The final DMSO concentration was maintained at 0.1% in all dilutions. Medium from DPX-2 cells in 96-well plates was aspirated using an EL406 liquid handler (BioTek); plates were then placed in the Precision XS, and 100 μL dosing medium containing the appropriate concentration of agonist/antagonist was transferred. Each condition was repeated in quadruplicate. After 24 hr, the cell medium was aspirated and cells were redosed as described above. After 48 hr, the cell viability (CellTiter-Fluor) and reporter assay (Bright-Glo) was carried out according to the protocol provided by Promega (Madison, WI).

Log-normalized drug concentrations were fitted to dose–response curves for each compound tested [relative luminescence units (RLU)], normalized to control (DMSO-treated) cells (agonist mode) and as a percentage of RLU observed with 10 μM rifampicin (antagonist mode). Curves were fitted using a nonlinear regression model [variable slope (four parameters) equation] (GraphPad Prism, version 4.0a; GraphPad Software Inc., La Jolla, CA). In this assay, the standard agonist compound rifampicin had a mean EC_50_ of 1.99 μM.

## Results

### Sampling ToxCast compounds as potential PXR agonists using docking

In the present study we used molecular docking coupled with hybrid scoring strategies to select compounds for *in vitro* testing based on predicted hPXR activity. Our initial selection of 15 ToxCast compounds was supplemented with 13 compounds from a previous study (Lemaire et al. 2006) that were also included in the ToxCast data set (Table 2). Using the docking consensus classification, we correctly classified 15 of 28 (55.6%) molecules from this sample ToxCast data set. Of these 28 compounds, 12 were classified as agonists and 16 as nonagonists, based on the actual ToxCast data. Docking-based classification using GoldScore correctly predicted 8 of 12 agonists and 7 of 16 nonagonists. Using the hybrid scoring scheme, we correctly predicted 7 of 12 agonists and 8 of 16 nonagonists. Mancozeb was classified as a nonagonist in all docking models and was found to be a nonagonist in experimental studies, whereas butafenacil, permethrin, and β-cyfluthrin were all classified as agonists in both the docking and experimental methods based on our HepG2 data and the NCGC ToxCast data (Judson et al. 2010) [Table 2 and Supplemental Material, Figure 1 (doi:10.1289/ehp.1001930)]. Foramsulfuron and bensulfuron methyl were both weak agonists in our hPXR luciferase assay in HepG2 cells but were classed as nonagonists based on the U.S. EPA NCGC data generated using the DPX-2 cell line (Judson et al. 2010), a derivative of HepG2. These compounds may be hPXR agonists but have only weak activity at high concentrations. With high concentrations of compounds, hPXR activation may not be detected in some cell lines because of cellular toxicity or limited solubility of the compound(s). Similarly, using the hPXR DPX-2 agonist assay, we found generally good agreement with the hPXR HepG2 data, although *Z,E*-fenpyroximate appeared to be a weak hPXR agonist; the cell viability of this compound suggests it is also cytotoxic [median inhibitory concentration (IC_50_), 0.04; Table 2].

The standard agonist compound rifampicin had a mean EC_50_ of 1.99 μM in DPX-2 cells, which is lower than reported previously (Ekins et al. 2007) and closer to those reported in other cell assay systems in HepG2 [EC_50_, 400 nM (Hurst and Waxman 2004)] and CV-1 cells [EC_50_, 700–852 nM (Chrencik et al. 2005); EC_50_, 710 nM (Moore et al. 2000)]. The NCGC data (Judson et al. 2010) were from DPX-2 cells, which we would expect to yield results similar to our own using DPX-2 cells, but not to our data using HepG2 cells. For example, fenarimol was classified as a nonagonist based on the docking and hybrid scores, whereas it is an agonist according to the NCGC data (Judson et al. 2010) and our Bayesian classification. In the present study, the docking scores for this compound were very close to the cutoff scores and hence could not be effectively predicted. This is a limitation of classifying compounds based on docking scores. Hybrid scores, which are designed based on similarity-weighted docking scores, can resolve these limitations only when the test compound has high similarity to the cocrystallized ligand. Compounds such as fenbuconazole and difenzoquat metilsulfate were classified as agonists based on the high GoldScores but were classified as nonagonists in the hybrid scoring scheme because of their low similarity to their respective cocrystalized ligands. NCGC experimental data classified both these compounds as nonagonists (Judson et al. 2010).

### Comparing NCGC and docking data for all ToxCast compounds

Our results with the sample data set suggest that computational docking methods can be used as an effective strategy in prioritizing future ToxCast compounds for *in vitro* testing as PXR agonists before actual testing. To test this hypothesis, we docked all the compounds from the ToxCast database to all five hPXR crystal structures and scored the complexes using GoldScore and hybrid scoring functions. Based on the *in vitro* NCGC ToxCast data (Judson et al. 2010), 65 compounds have been classified as agonists of hPXR and 246 as nonagonists (U.S. EPA 2010). In the present study, GoldScore-based classification correctly predicted 48 agonists and 120 nonagonists, whereas the hybrid scoring scheme correctly predicted 45 agonists and 137 nonagonists [Supplemental Material, Table 2 (doi:10.1289/ehp.1001930)]. The rest of the compounds were either false positives or false negatives. The sensitivity of scoring schemes for correctly predicting hPXR agonists with the NCGC data (Judson et al. 2010) was 73.8% and 69.2% for GoldScore and hybrid score classification schemes, respectively.

### Predictions using the steroidal Bayesian hPXR model

The hPXR agonist predictions using the Bayesian model based on steroidal compounds (Ekins et al. 2009) classified all compounds other than mancozeb as agonists, based on the specified activity cutoff (Table 2).

### Testing for hPXR antagonists and allosteric antagonists

We evaluated whether some of the molecules selected from ToxCast by docking (mesosulfuron-methyl, bensulfuron methyl, esfenvalerate, *Z,E*-fenpyroximate, α-cypermethrin, β-cyfluthrin, and permethrin) were also potential PXR antagonists or allosteric antagonists (Ekins et al. 2007, 2008a) in the presence of rifampicin. Our results for these compounds tested with the DPX-2 cell assay suggest that they were not antagonists or allosteric antagonists (data not shown).

## Discussion

Recent studies have showed the complexity and challenges of producing predictive computational models for hPXR (Ekins et al. 2009). In the present study we used docking to analyze the ToxCast compounds and select molecules to validate the approach of identifying compounds of interest from large data sets without experimentally screening the whole data set. Subsequently, the release of the NCGC data in which the whole data set was experimentally screened provided additional data to compare with our own laboratory hPXR activation data and our computational predictions in five crystal structures. An advantage of using these different cell systems is that some are more sensitive than others and thus may identify additional compounds as agonists compared with using a single cell type (Table 2). For example, diethylhexyl phthalate, esfenvalerate, α-cypermethrin, β-cyfluthrin, and permethrin were more active in HepG2 cells compared with NCGC DPX-2 cells (Table 2). Fenarinol, imazalil, alachlor, and fipronil were classed as active in NCGC DPX-2 cells but not in HeLa cells [as reported previously by Lemaire et al. (2006)], whereas fenbuconazole, and prochloraz were more active in HeLa cells than DPX-2 cells (Table 2) (Lemaire et al. 2006).

Possible reasons for these differences include non–liver-type cells possibly lacking transporters (or expressing different ones) that can influence drug entry/efflux or may show differential toxicity. This is a problem with conjugated steroids and bile salts. We are not sure if this is the case for the pesticides and industrial chemicals studied here. HeLa cells [used by Lemaire et al. (2006)], HepG2 cells, and hepatocytes are markedly different in terms of their cells of origin (HeLa and HepG2 are essentially cancer cell lines). The HeLa cell line may have different levels of corepressors and coactivators that affect the function of PXR differently than in liver cell lines. The transactivation of reporters varies in both cell lines to a degree. For example, HNF4α-mediated effects on PXR are greater in HeLa cells than in HepG2 cells, suggesting weaker PXR transactivation profiles in HeLa versus hepatic-derived cell lines (Tirona et al. 2003). One could perhaps use primary human hepatocytes for such studies, but they have the limitations of cost, limited supply, and variability. It is also possible that cell lines differentially metabolize the test compounds, which could also affect PXR activation results. In the pressent study (and for the NCGC data), the metabolism of the compound in the *in vitro* assay was not analyzed. Because radioligand binding is not a viable option for high-throughput screening for PXR and additionally would probably not give information on actual overall activity, there may be differences between docking and functional assays, a situation likely not unique to PXR.

All compounds from the ToxCast data set were docked to the five hPXR crystal structures and scored using GoldScore and hybrid scoring schemes. When molecular docking is used as a tool for classification studies, the question arises as to what docking scores should be considered as a cutoff. Because there are no standard rules governing the choice of cutoff scores, in the past we have designed hybrid scoring schemes that used similarity scores derived from molecular shapes as a weighting factor (Kortagere et al. 2009). The goal of the hybrid scoring schemes is to increase the gap between hPXR agonists and hPXR nonagonists and thus ease classification of compounds. In this study we derived cutoff scores either by using the docking scores of the cocrystal structure ligands or by averaging the docking or weighted scores of all the ligands binding to the five crystal structures (Table 1). Predictions from the complete ToxCast data set performed marginally better than the sample data set, thereby emphasizing the utility of the approach to classify large data sets prior to *in vitro* testing. The hybrid scoring scheme had a sensitivity of approximately 69%, specificity of approximately 56%, overall prediction accuracy of approximately 59%, and a Matthews correlation coefficient of 0.2 for classifying all ToxCast compounds based on the NCGC data (Judson et al. 2010), whereas the GoldScore-based scheme provided the best sensitivity of approximately 74% (Table 3). Classification studies also benefit from the availability of multiple crystal structures of the protein in complex with a variety of ligands. Although all the cocrystallized ligands bind to the same binding site and the crystal structures superimpose within approximately 1 Å root mean squared deviation, the size, nature, and chemical composition of these ligands are very different (Ekins et al. 2009). In addition, the availability of multiple crystal structures of promiscuous proteins such as PXR helps improve sampling of the docking mode of these compounds. This is evident in the range of docking scores we obtained for each compound across each of the five crystal structures [see Supplemental Material, Table 2 (doi:10.1289/ehp.1001930)], which also emphasizes that averaging methods to get a single score should not be used in these cases. Instead, we classified each docked complex using the cutoff scores listed in Table 1 and then used a majority vote (3 of 5) method for consensus classification for both GoldScore and hybrid scoring schemes.

The ToxCast data set also consists of compounds that have diverse structure, size, and chemical composition, albeit with similar functionality important for interactions with PXR [see Supplemental Material, Figure 1 (doi:10.1289/ehp.1001930)]. Thus, classifying this diverse data set by similarity-weighted scoring schemes with approximately 69% sensitivity for predicting agonists is encouraging (considering the promiscuity of the receptor), and the statistics are comparable with or better than our previous data from studies using the GoldScore and hybrid scoring schemes (Ekins et al. 2009; Kortagere et al. 2009). These data should improve in the future as docking tools develop; however, the current approaches may be more accurate with other less-promiscuous nuclear receptors such as the estrogen and androgen receptors. The hybrid scoring scheme performed better for the ToxCast data (sensitivity and Matthews correlation coefficient were consistently higher; Table 3) compared with a series of 119 steroidal molecules we previously used with average sensitivity of 52%, specificity of 50.34%, accuracy of 50.76%, and Matthews correlation coefficient of 0.02% (Kortagere et al. 2009). This suggests that there may be differences in docking utility depending on compound class and therefore value in evaluating molecules beyond drugs to gain a broader insight into potential PXR agonists among industrial chemicals, pollutants, natural products, and so on.

We also compared docking with a ligand-based QSAR method based on steroidal compounds to provide a further benchmark, and in this case its performance was poor, possibly for several reasons. First, many groups have illustrated the importance of chemical space coverage and the applicability domain of ligand-based models (Chekmarev et al. 2008, 2009; Dimitrov et al. 2005; Ekins et al. 2006; Kortagere et al. 2008, 2009; Sheridan et al. 2004; Tetko et al. 2006, 2008). When we analyzed the molecular space covered by the steroids in the ligand-based Bayesian model compared with the ToxCast compounds, we found that they can be clearly separated [see Supplemental Material, Figure 2 (doi:10.1289/ehp.1001930)], which is indicative of little overlap. This would suggest it may be difficult for this local hPXR model to reliably predict compounds that are not steroidal. Thus, it may be important in future work to develop a separate Bayesian model with the ToxCast compounds to predict hPXR agonists added to later versions of the database. Our ligand-based data also confirm that to predict diverse compounds with likely hPXR agonist activity, methods that are more generic or global in nature are required that can capture some of the flexibility of the ligand-binding domain (Ekins et al. 2009). Although ToxCast is a major U.S. EPA initiative for prioritizing toxicity testing of large numbers of pesticides and other chemicals (Dix et al. 2007; Houck et al. 2009; Judson et al. 2009, 2010; Knight et al. 2009), we suggest that it also represents a unique opportunity to evaluate various predictive computational approaches used for toxicology end points prospectively in addition to the many ways to mine the data retrospectively. In the present study we have addressed only a tiny fraction of the data produced to date and perhaps raised the question that focusing on one cell line for a single nuclear receptor, such as hPXR, may be too simplistic.

We still can learn a great deal from the efficient combination of *in vitro* and *in silico* approaches, such that multiple iterations of prediction may yield a more cost-effective route to selecting compounds for testing from a large database. The ToxCast data set therefore represents an important and evolving basis for evaluating computational methods used in toxicological assessments of compounds important for environmental and health applications. Although we did not identify any PXR antagonists or allosteric antagonists, based on the few samples tested, the complete ToxCast data set could be more exhaustively studied with both computational and *in vitro* methods in the future (Ekins et al. 2008a). Current opinion suggests that classical competitive antagonists for PXR that bind in the ligand-binding pocket may be difficult to identify (Xue et al. 2007a) compared with allosteric antagonists that bind elsewhere on the protein surface (Ekins et al. 2007, 2008a). It is therefore important to evaluate whether a compound may be a more selective allosteric antagonist, because this could outweigh any potential PXR agonist activity *in vivo*.

## Figures and Tables

**Table 1:** GoldScores for the PXR cocrystallized ligands and their corresponding cutoff scores used for classification of a sample data set and all ToxCast compounds using GoldScore and 2D similarity-weighted hybrid score.

PXR structure (PDB code)	Cocrystallized ligand		Cutoff score for sample data set		Cutoff score for ToxCast data set	
	PDB code	GoldScore	GoldScore	Hybrid score	GoldScore	Hybrid score
1M13	HYF	82.06	66	15	51	13
1NRL	SRL	48.31	39	15	46	14
1SKX	RFP	65.44	52	15	48	15
2O9I	444	48.69	39	15	46	13
2QNV	CDZ	55.19	44	15	44	11

**Table 2:** Summary of predicted and experimental data for sample ToxCast data set compounds.

Compound	Docking classification^a^	Bayesian score (classification)	U.S. EPA ToxCast (NCGC) hPXR DPX-2 classification [EC_50_ (μM)]^b^	HepG2 hPXR EC_50_ (μM)^c^	Efficacy relative to 10 μM rifampicin^c^	Cell viability IC_50_ (μM)^c^	DPX-2 hPXR EC_50_ (μM)^c^
Mancozeb	N	−7.253 (N)	N	X			
Mesosulfuron-methyl	A	−1.589 (A)	N	X		X	X
Diethylhexyl phthalate	A	1.943 (A)	A (20.75)	1.8 (S)	0.63		
Methyl hydrogen phthalate	N	1.868 (A)	N				
Bensulide	A	−1.165 (A)	A (1.57)				
Foramsulfuron	A	−1.653 (A)	N	> 50 (W)			
Bensulfuron methyl	A	0.601 (A)	N	89.4 (W)	0.18	X	X
Esfenvalerate	A	5.796 (A)	A (26.98)	1.5 (S)	0.64	X	8.94 (S)
*Z,E*-fenpyroximate	A	2.613 (A)	N	X		0.04	32.74 (M)
Butafenacil	A	3.317 (A)	N	6 (S)	0.53		
α-Cypermethrin	A	5.346 (A)	A (18.3)	1.6 (S)	0.54	X	0.88 (S)
Triflusulfuron methyl	A	−1.998 (A)	N	–			
β-Cyfluthrin	A	5.346 (A)	A (19.7)	2.5 (S)	0.54	> 100	18.2 (M)
Permethrin	A	4.83 (A)	A (20.26)	5.4 (S)	0.53	X	29.09 (M)
Oxasulfuron	A	−1.942 (A)	N				
Fenarimol^d^	N	4.791 (A)	A (20.29)				
Propiconazole^d^	A	3.475 (A)	A (36.81)				
Fenbuconazole^d^	A	5.390 (A)	N				
Prochloraz^d^	A	2.705 (A)	N				
Imazalil^d^	N	3.466 (A)	A (36.54)				
Oxadiazon^d^	A	4.663 (A)	A (5.49)				
Alachlor^d^	N	7.842 (A)	A (15.35)				
2,4-D^d^	N	−0.563 (A)	N				
Diuron^d^	N	4.357 (A)	N				
Atrazine^d^	N	−2.825 (A)	N				
Fipronil^d^	N	−0.033 (A)	A (12.55)				
Thiabendazole^d^	N	2.879 (A)	N				
Carbaryl^d^	N	1.265 (A)	N				

Abbreviations: 2,4-D, 2,4-dichlorophenoxyacetic acid; A, agonist; M, medium agonist; N, nonagonist; S, strong agonist; W, weak agonist; X, no activity measurable. Values with Bayesian scores greater than −5.792 were classed as PXR agonists based on the model output (Ekins et al. 2009). Docking classification was performed using GoldScore, with cutoff values listed in Table 1. Agonists were classified based on the following criteria used in a previous study (Ekins et al. 2008b): S, EC_50_ < 10 μM; M, EC_50_ 11–50 μM; W, EC_50_ > 50 μM (but with activation at least 10% that of 10 μM rifampicin).

a Based on data provided in Supplemental Material, Table 1 (doi:10.1289/ehp.1001930).

b For NCGC data (Judson et al. 2010), the cutoff for activity was 200 μM.

c Assays performed in the present study.

d Componds with previously published data generated in HeLa cells (Lemaire et al. (2006).

**Table 3:** Statistical parameters for the hPXR consensus docking results from the sample data set (*n* = 28) and complete (all; *n* = 308) ToxCast data set.

Data set	SE	SP	Q	C
Sample data set, gs	66.67	46.67	55.55	0.13
Sample data set, hs	58.33	50.00	53.57	0.08
All ToxCast, gs	73.85	51.22	55.95	0.20
All ToxCast, hs	69.23	55.69	58.52	0.20

Abbreviations: C, Matthews correlation coefficient; gs, GoldScore; hs, hybrid scoring schemes; Q, overall prediction accuracy; SE, sensitivity; SP, specificity.
